# Eucalypt pulp yield QTL from Raiz as compared to the literature

**DOI:** 10.1186/1753-6561-5-S7-P48

**Published:** 2011-09-13

**Authors:** Cristina MP Marques, Victor J Carocha, Carla Ribeiro, Fátima Cunha, António Mendes de Sousa, José A Araújo, João Costa e Silva, José Carlos Rodrigues, Ana Freitas, Ana M Pires, Gabriel Dehon Rezende

**Affiliations:** 1 RAIZ, Portugal; 2 ITQB, Portugal; 3 CBA/DBA, FCUL, Portugal; 4 CEF, ISA/UTL, Portugal; 5 IICT, Portugal; 6 DM-CEMAT, IST/UTL, Portugal

## Background

RAIZ is a Portuguese private non-profit research institute owned by the Pulp & Paper Portucel Soporcel Group (http://www.raiz-iifp.pt). RAIZ *E. globulus* genetic improvement program is managed in order to generate trees with increased economic value, through gains in forest productivity and wood properties. Molecular markers have been used for clonal identification and genetic diversity management in the program since 1990. Moreover, RAIZ has been engaged in a longer term genomics project aiming to identify quantitative trait *loci* (QTL) for wood properties. The ability to detect QTL depends on sample size, genetic background, environment and genetic interactions. Most importantly, the ability to use detected QTL depends on their adequate map location and the identification of the molecular variation behind them. This in turn is determined by linkage map quality, the choice of phenotypes and the precision of phenotyping. There are many reports in the literature on QTL detection for economically important traits in *Eucalyptus*, but very few present data on QTL verification (at the statistical and/or biological levels). We illustrate the importance of this issue for pulp yield related traits by comparing available results from QTL mapping studies in the literature with those obtained from a QTL detection and verification experiment pursued by RAIZ.

## Methods

An F_1_ full-sib family with 361 progeny from an intraspecific *E. globulus* cross was planted in a field trial and phenotyped (at age 4_1/2_ years) for pulp yield using near infrared spectroscopy. Phenotypic data was adjusted for spatial variation in the field trial using a first-order separable autoregressive model. The NIR-PLSR spectra were recorded using a *Bruker* equipment (*vector 22N model*), and the calibration model to estimate pulp yield (RTIK16) was constructed using partial least-squares regression as implemented in with the *QUANT2 software*. DNA extraction and microsatellite genotyping were carried out as described in [1,2]. *Linkage* analysis and map construction were performed on independent male and female datasets using *MAPMAKER/EXP® 3.0*[3] under the F_2_ backcross model. Framework maps with evenly distributed selected SSR and gene markers were used for QTL detection [4-8]. The selection of SSR markers took into consideration the possibility to establish synteny between available eucalypt maps in the literature. Interval Mapping and Multiple Interval Mapping QTL detection results were compared with the *MultiQTL vs 2.6 software*. QTL detection was repeated in data from 100 different simulations (using the *R software*), after adding to the original phenotypes randomly selected values from a normal distribution with mean zero and a standard deviation that accounted for the reference essay error and the prediction error of the NIR calibration model.

## Results

**Figure 1:**
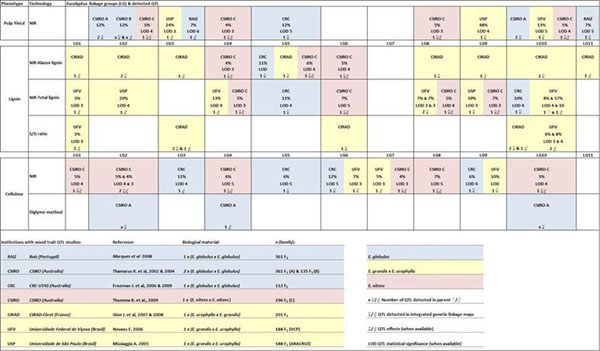
Raiz and literature QTL studies for pulp yield related traits in *Eucalyptus*, compared at the linkage group (chromosome) level

Many QTL for pulp yield related traits have been reported for every eucalypt chromosome, in the literature. There is insufficient mapping information to infer if these QTL where detected in similar genomic locations. In 6 of the 11 eucalypt linkage groups (LG2, LG4, LG5, LG6, LG9 and LG10), QTL for pulp yield related traits were detected in different species (*E. globulus*, *E. grandis* x *E. urophylla* and/or *E. nitens*) (Figure 1). RAIZ reports two verified QTL for pulp yield, in an *E. globulus* intraspecific cross, in linkage groups 3 and 11, detected in all simulations. Although these results can reflect the polygenic nature of the trait (as suggested by [9]) we cannot exclude the putative biases resulting from current limitations of existing studies in terms of experimental designs, phenotyping and/or data analysis.

## Conclusions

QTL studies in some plant species have proved useful to target genomic regions for subsequent genomics investigation [10]. Forest trees experience a variety of environmental conditions and it is expected that some QTL will be age/environment specific and some will be consistently detected over multiple growing seasons. In order to raise the scope of inferences that can be drawn from QTL research in *Eucalyptus* and the prospects of delivering breeding tools from gene sequences, future QTL detection studies should account for experimental phenotyping error, in order to reduce putative false positive results, as we have done in RAIZ QTL detection experiment. Existing QTL mapping studies from different institutions could be upgraded in this perspective, in order to allow comparative mapping and the identification (or not) of specific genomic regions, detected in multiple *pedigrees* and environments, that could be explored further.
